# Current News

**Published:** 1889-08

**Authors:** 


### Current News.

Dr. Guilford's work on Orthodontia is now in preparation, and will be out in time for the opening of the college year. The book will be amply illustrated, and reach upward of two hundred pages. From examination of advance sheets we can heartily recommend it to students and practitioners.

---

The National Association of Dental Faculties voted, at Saratoga, to extend the course of instruction to three years, to take effect at the beginning of the session of 1891–92.

---

The sixth annual meeting of the Minnesota State Dental Association was held at the Spalding House, Duluth, July 10–12. There was a good attendance and an interesting programme. By way of entertainment the Duluth dentists gave the visitors a boat-ride on the lake, and Dr. Brown gave a reception at his residence.

The officers for the ensuing year are Dr. G. O. I. Brown, Duluth, President; Dr. L. C. Davenport, Moorhead, Vice-President; Dr. C. H. Robinson, Wabasha, Recording Secretary; Dr. M. G. Jenison, Minneapolis, Corresponding Secretary; Dr. H. M. Reid, Minneapolis, Treasurer.

The next annual meeting will be in Minneapolis, commencing the second Wednesday in July, 1890.

Very truly,

M. G. Jenison.

---

The Connecticut Valley Dental Society, the New England Dental Society, and the Connecticut State Dental Society will unite in a union meeting at Springfield, Mass., commencing Wednesday, October 23, and continuing through the 24th and 25th. Prominent members of the profession will read interesting papers, and, as special arrangements will be made for viewing the clinics, they will therefore prove more than usually instructive.

A cordial invitation is extended to members of dental societies.

Per order of joint committees.

George A. Maxfield, D.D.S., *Secretary.*

